# Comparison of clinical characteristics, hospital treatment and outcomes in all four waves of Covid-19 patients at RTEH Muzaffargarh, Pakistan

**DOI:** 10.12669/pjms.40.2(ICON).8947

**Published:** 2024-01

**Authors:** Masood Alam, Aneel Razzak, Muhammad Hussain Shah Gilani, Zahid Siddique Shad, Sheraz Hassan

**Affiliations:** 1 Dr. Masood Alam, MBBS, FCPS (Pulmonology). Department of Consultant Pulmonology, Recep Tayip Erdogan Hospital, Muzaffargarh, Pakistan; 2 Dr. Aneel Razzak, MBBS, FCPS (Pulmonology). Consultant Pulmonology Department, Recep Tayip Erdogan Hospital, Muzaffargarh, Pakistan; 3 Dr. Muhammad Hussain Shah Gilani, MBBS, FCPS (Medicine). Consultant Medicine Department, Recep Tayip Erdogan Hospital, Muzaffargarh, Pakistan; 4 Dr. Zahid Siddique Shad, MBBS, FCPS. Department of Consultant ICU, Recep Tayip Erdogan Hospital, Muzaffargarh, Pakistan; 5 Dr. Sheraz Hassan, MBBS, FCPS (Medicine). Department of Consultant Medicine, Recep Tayip Erdogan Hospital, Muzaffargarh, Pakistan

**Keywords:** Covid19 pneumonia, Pandemic Waves, Complications, Outcome, Treatment

## Abstract

**Background and Objective:**

The coronavirus pandemic followed a succession of COVID-19 waves globally, and had a varying pattern of frequency of cases and disease spectrum as each wave came with its distinct viral characteristics. The objective of this study was to compare clinical characteristics, treatments and outcomes of patients admitted with severe COVID -19 pneumonia in all four waves at Recep Tayyip Erdogan Hospital (RTEH).

**Methods:**

A cross sectional retrospective study was conducted at the COVID unit of Recep Tayyip Erdogan Hospital (RTEH), Muzaffargarh, from April 2020 to December 2021. Retrospective data was taken from Electronic Medical Records of patients of Covid pneumonia and divided into four groups according to four waves of Covid pandemic. The main objective was to compare disease spectrum, treatments and outcomes of patients admitted with severe COVID-19 pneumonia in all four waves at RTEH. Demographic characteristics, inflammatory markers such as C reactive protein (CRP), serum lactate dehydrogenase (LDH), serum ferratin and absolute lymphocyte counts, mortality, length of hospital and ICU stay and event of mechanical ventilation were compared between groups. The Kolmogorov-Smirnov test was applied to check the normality. P-value <0.05 was considered significance.

**Results:**

Of a total of 903 patients with covid pneumonia, 521(57.7%) were males and 382 (42.3%) females. Their mean age was 55.56±15.06 years. The mean length of stay (LOS) at the hospital was higher in first wave and least in fourth wave, 9.06±6.46 days and 6.56±5.34 days, respectively, (p<0.010). In first wave, LOS was generally >10 days with 21 (22.6%) while 33(26.8%) patients were shifted to ICU in first and second waves, respectively. Whereas, 35(8.2%) patients shifted to ICU in fourth wave (p<0.010). The use of mechanical ventilation was most common in first and second wave, 14 (15.1%) and 18 (14.6%), respectively. Mortality rate was highest in the third wave, 102 (38.9%, p<0.010) compared to the rest of the waves.

**Conclusion:**

Comparison of COVID-19 pneumonia patients across pandemic waves has revealed dynamic trends in patient outcomes. The initial waves had higher ICU admissions and mortality rate, suggesting a need for improved early response and resource allocation. Continuous adaptability in healthcare strategies was paramount for enhancing patient care during the ever-changing pandemic landscape.

## INTRODUCTION

Coronavirus disease 2019 (COVID-19) caused by a novel coronavirus, named severe acute respiratory syndrome coronavirus-2 (SARS-CoV-2) originated in Wuhan, Hubei Province, Central China and had a worldwide effect since it was designated a pandemic on March 11, 2020.^1^ Till now, the World Health Organization (WHO) has documented 136,996,364 confirmed COVID-19 cases, with 2,951,832 fatalities.^2^ It has wreaked havoc on the healthcare systems all over the world since the announcement of this worldwide pandemic.^3^ Although COVID-19 may be asymptomatic, it may cause severe pneumonia-like symptoms. International standards have previously suggested dexamethasone and remdesivir as treatment options. Nonetheless, mortality rates, continue to be high.^4^

Viruses gradually change over time, including SARS-CoV-2, the virus that caused COVID-19. Thus, in late 2020, World Health Organization classified some variants as variants of interest (VOIs) and variants of concern (VOCs) in order to prioritize global monitoring and research as well as to educate and modify the COVID-19 response. These variances increased the risk to public health on a global scale.^4^ B.1.1.7 (alpha), B.1.351 (beta), P.1 (gamma), and B.1.617.2 (delta) are four unique viral lineages that have appeared globally and should be closely monitored.^5^ The most prominent variations of concern lately were alpha, beta, and delta variants, which contributed greatly to global increase of new waves.

Treatment regimens have evolved in different waves of Covid-19 pandemic. Before the global roll on of its second wave, three important developments to counterfiet Covid-19 and its acute sequelae emerged. Firstly, as a response to the remarkably high incidence of thrombotic complications, guidelines were rapidly adjusted to address increased awareness and proper diagnosis of VTE, and adapt dosage of low-molecular-weight heparin (LMWH) thromboprophylaxis.6-9 Second, remdesivir administered to hospitalized patients with COVID-19 was suggested as superior to placebo in shortening recovery time in adults.^10^ Third, dexamethasone was demonstrated to reduce mortality in critically ill Covid-19 patients.^11^ For patients on ventilators, dexamethasone was shown to reduce mortality by about one third, and in patients requiring only oxygen for recovery, mortality reduced by about one fifth.^10^ Recommendations came against the use of hydroxychloroquine, azithromycin and convalescent plasma.^12^

In the continuing pandemic, Corona virus kept on mutating with reporting of different strains up-till now. Thus, treatment evolved from the first wave of COVID to date. This study aimed to comprehensively analyses clinical and treatment aspects of COVID-19 across multiple pandemic waves; focusing on a specific geographic region. This localized and retrospective study would allow us to uncover distinctive trends and variations in patient care and outcomes, offering valuable insights for tailored pandemic response strategies.

## METHODS

This retrospective cross-sectional observational research was conducted in hospitalized patients diagnosed with COVID-19 pneumonia in RTEH from April 2020 to December 2021. The study population included patients of both genders from 18 years to 80 years of age with severe COVID 19 pneumonias. Patients who left against medical advice during treatment or had previous history of admission in COVID units in any other hospital were excluded from the study. Retrospective data of all 903 patients from April 2020 to December 2021 fulfilling the inclusion criteria was included. As there was no active intervention in this trial, informed consent was not required but data confidentiality was dealt with in accordance to ethical norms.

### Ethical Approval

It was obtained from IHHN’s IRB, IHHN_IRB_2022_10_010,

Using a predetermined case report form, the hospital’s Electronic Medical Records were utilized to collect and record important clinical and laboratory data. Age, sex, comorbidities, and medication details of the patients were documented. Medical record of each patient was tracked for PCR status of COVID (negative/positive) in hospital treatment, history of steroid, vaccine status, inflammatory markers on admission, ICU admission, and mechanical ventilation.

Patients were divided into four groups according to the four waves of Covid pandemic. Demographic characteristics, inflammatory markers such as C-reactive protein (CRP), Lactate dehydrogenase (LDH) and serum ferratin and absolute lymphocyte counts, mortality rate, length of hospital stay and ICU stay and event of mechanical ventilation were compared between each group.

### Statistical Analysis

SPSS 22 was used to analyze data. Age, absolute lymphocyte counts, LDH, CRP, serum ferritin levels, duration of mechanical ventilation, CT scan severity score and length of hospital stay were all quantitative variables that were statistically analyzed in mean and standard deviation. To check the significance, kruskal-Wallis H Test was applied. Gender, comorbidities, clinical severity on admission, admission in ICU, mechanical ventilation, complications, Covid PCR status, and death or discharge were analyzed in percentages and frequencies. Chi-square test was applied to check significance and P-value < 0.05 was considered significant. The Kolmogorov-Smirnov test assessed normality.

## RESULTS

A total of 903 patients were admitted in RTEH, Muzaffargarh, in Covid-19 ward in all four waves. The mean age of the patients was 55.56±15.06 years. There were 521(57.7%) males and 382(42.3%) females. Only 18(2.0%) of the patients were current smokers.

Of these, 93 (10.3%) were treated in the first wave, 123 (13.6%) in second wave, 262 (29.0%) in third wave and 425 (47.1%) in the fourth wave. More females than males’ patients were treated in the first wave 70 (75.3%) and second waves 86 (69.9%), respectively, while, there were very few males in the first wave 00(00%) (p=0.000).

Patients throughout the four waves were mostly >30 years of age although the first wave received more patients of <30 years, (p=0.000). Table-I The mean TLC, CRP, LDH, absolute lymphocytes and ferritin on admission of all the patients was 11.55±12.50, 81.06±67.79, 748.75±641.08, 942.62±782.09 and 1352.68±1272.86, respectively. The mean TLC and CRP were lower in first wave than other three waves. LDH was lowest in fourth wave, absolute lymphocytes were lowest in first wave and ferritin was lowest in third wave, (p<0.010). The differences between TLC, CRP, LDH, absolute lymphocytes and ferritin on admission in all four waves were statistically significant, (p<0.010). Very sever stage of disease was maximum (35.1%) in third wave and also mild severity of disease was minimum 5(1.9%) in third wave (p=0.000).

**Table-I:** Demographic and baseline characteristics of the COVID-19 patients in four waves.

Variable	First, N (%)	Second, N (%)	Third N (%)	Fourth N (%)	P-value
***Gender***					
Male	23 (24.7)*	37 (30.1)	108 (41.2)	214 (50.4)	0.000
Female	70 (75.3)*	86 (69.9)*	154 (29.6)	211 (49.6)*	0.000
***Age***					
Mean ± SD	51.01±16.71	52.68±15.78	56.58±13.4	56.76±15.1	0.000
<30 year	16 (17.2)	13 (10.6)	610 (3.8)	617 (4.0)	0.000
30-60 years	46 (49.5)	68 (55.3)	144 (55.0)	225 (52.9)	0.000
60 years	31 (33.3)	42 (34.1)	108 (41.2)	183 (43.1)	0.000
***Smoking status***					
Current smoker	7(7.5)	0(0.0)	3(1.1)	8(1.9)	0.000
Ex-smoker	6(6.5)	2(1.6)	14(5.3)	15(3.5)	0.001
Non smoker	80 (86.0)	121 (98.4)	245 (93.5)	402 (94.6)	0.000
***TLC on admission (cell/cubic mm)***					
Mean=SD	8.64±9.05	12.81±14.79	12.08±14.70	11.50±10.77	0.000
***CRP on admission***					
Mean=SD	68.21±59	117.84±59.84	78.37±62.47	74.89±71.46	0.000
LDH on admission					
Mean =SD	854.55±83.2	927.07±46.5	961.91±529	542.58±628	0.000
***Absolute lymphocytes on admission***					
Mean=SD	544.95±679.1	1079.88±744.1	1032.02±769	934.81±795	0.000
***Ferritin on admission***					
Mean=SD	1531.69±1347	1371.85±1294	1094.41±107	1504.92±1358	0.000
***Clinical stage of disease***					
Mild	33 (35.5) *	4 (3.3)	5 (1.9) *	33 (7.8)	0.000
Moderate	3 (3.2) *	22 (17.9)	42 (16.0)	40 (9.4)	0.000
Severe	36 (38.7)	60 (48.8)	123 (46.9)	208 (48.9)	0.000
Very Severe	21 (22.6)	37 (30.1)	92 (35.1)	144 (33.9)	0.000
***Pre-hospital treatment***					
Antibiotics	24 (25.8)	25 (20.3)	43 (16.4)	173 (40.7)*	0.000
Remdesivir	4 (4.3)	48 (39.0)	186 (71.0)*	235 (55.3)	0.000
Steroids	5 (5.4)	2 (1.6)	5 (1.9)	0 (0.0)	0.000
Anticoagulant	3 (3.2)	3 (2.4)	3 (1.1)	0 (0.0)	0.000
None	57 (61.3)	45 (36.6)	25 (9.5)	17 (4.0)	0.000
***Covid-19 PCR status***					
Positive	60 (64.5)	110 (89.4)	238 (90.8)	344 (80.9)	0.000
Negative	33 (35.5) *	13 (10.6)	24 (9.2)	81 (19.1)	0.000
***Vaccination status***					
Completed	-	-	1 (0.4) *	33 (7.8) *	0.000
Partial complete	-	-	3 (1.1)	38 (8.9) *	0.000
No	93 (100.0)	123 (100.0)	258 (98.5)	354 (83.3)	0.000

Antibiotics were most commonly used before reaching hospital (pre- hospital in all four waves while remdesivir was most commonly used in third wave, (p=0.000). Regarding vaccination status, one (0.4%) patient was fully vaccinated in third wave and 33 (7.8%) in fourth wave, (p=0.000), (Table-I).

Diabetes mellitus and hypertension were seen in 347 (38.4%) and 118 (13.1%) patients, but the distribution of diabetes and hypertension was almost equal in all four waves, (p=0.622). (Table-II). Majority of the patients were non-smokers while more smokers were in the first wave, (p=0.001).

**Table-II:** Comorbidity of the patients.

COMORBIDITIES	COVID 19-WAVES				P-value

	First, N (%)	Second, N (%)	Third N (%)	Third N (%)	
Asthma	3 (3.2)	1 (0.8)	7 (2.7)	7 (1.6)	0.622
Bronchiectasis	0 (0.0)	0 (0.0)	3 (1.1)	2 (0.5)	
Chronic kidney disease	0 (0.0)	1 (0.8)	3 (1.1)	3 (0.7)	
Chronic liver disease	0 (0.0)	1 (0.8)	1 (0.4)	2 (0.5)	
COPD	2 (2.2)	1 (0.8)	6 (2.3)	13 (3.1)	
Diabetes mellitus	29 (31.2)	57 (46.3)	97 (37.0)	164 (38.6)	
Hypertension	10 (10.8)	17 (13.8)	37 (14.1)	54 (12.7)	
Ischemic heart disease	4 (4.3)	5 (4.1)	11 (4.2)	8 (1.9)	
No disease	45 (48.4)	40 (32.5)	97 (37.0)	172 (40.5)	
Asthma	3 (3.2)	1 (0.8)	7 (2.7)	7 (1.6)	

The LOS was highest in first wave while least in fourth wave as 9.06±6.46 days and 6.56±5.34 days, respectively, (p=0.000). Length of hospital stay was >10 days as standardized residual ≥1.96 and p=0.020 in first wave. Patients requiring treatment in ICU was 21(22.6%) in first wave, 33(26.8%) in second wave, 44(16.8%) in third wave and 35 (8.2%) in fourth wave, (p=0.000). Similarly, the use of mechanical ventilation was most common in first 14 (15.1%) and in second 18 (14.6%) wave, while it was least in fourth wave 21 (4.9%), (p=0.000). Mortality was highest, 102 (38.9%) in third wave, (p=0.000) compare to the rest of the waves. The Kolmogorov-Smirnov test showed that the variables, LOS and ICU stay were not normal, (p<0.010), (Table. III).

**Table-III:** Presentation of outcomes in the study groups

Variables	Covid-19 wave				P-value

	First, N (%)	Second, N (%)	Third N (%)	Fourth N(%)	
***Length of hospital stay (Days)***					
Mean ± S.D.	9.06±6.46	7.42±6.01	7.22±5.88	6.56±5.34	0.000
1-5	34 (36.6)	62 (50.4)	139 (53.1)	222 (52.2)	0.020
6-10	32 (34.4)	34 (27.6)	75 (28.6)	139 (32.7)	
>10	27 (29.0) *	27 (22.0)	48 (18.3)	64 (15.1)	
***ICU admission***					
Yes	21 (22.6) *	33 (26.8) *	44 (16.8)	35 (8.2) *	
No	72 (77.4)	90 (73.2)	218 (83.2)	390 (91.8)	0.000
***Length of ICU stay***					
Mean ± S.D.	9.38±5.55	8.69±6.23	9.07±7.22	7.68±5.92	0.000
***Mechanical Ventilation***					
Yes	14 (15.1) *	18 (14.6) *	18 (6.9)	21 (4.9) *	
No	79 (84.9)	105 (85.4)	244 (93.1)	404 (95.1)	0.000
***Final outcome***					
Death	21 (22.6)	39 (31.7)	102 (38.9) *	145 (34.1)	0.000
Discharge at room Air	66 (71.0)	82 (66.7)	132 (50.4)	246 (57.6)	
Discharge to home	6 (6.5)	2 (1.6) *	28 (10.7)	34 (8.0)	

* Standardized residual ≥1.96, P≤0.05 considered as significant, S.D.: standard deviation, C.I: confidence interval, ICU: intensive care unit

## DISCUSSION

The COVID-19 pandemic was an unprecedented global health crisis, necessitating continuous adaptation of healthcare strategies to address the evolving landscape of the disease. Our study, conducted at RTEH, Muzaffargarh over four distinct pandemic waves, provides valuable insights into the changing profile of COVID-19 patients and their clinical outcomes. The mean age of our study cohort, 55.56 years, is consistent with previous reports highlighting the increased vulnerability of older individuals to severe COVID-19 outcomes 13. Notably, we observed variations in gender distribution across different waves, with early waves having a higher proportion of females. This gender-related disparity warrants further investigation to determine underlying factors driving these shifts.^2^ The dominance of patients aged over 30 years in our study aligns with global trends. However, the first wave saw an intriguing reversal with a higher prevalence of patients under 30, indicating potential variations in susceptibility or behavior in this age group during the early stages of the pandemic.

Guo T et al.^14^ studied 105 elderly patients in China during the first wave of Covid19. Total patients included 81% middle aged population. They observed fewer complications among the younger group as compared to the elderly group (14.1% vs. 40.0%, p=0.0014). Invasive ventilatory support was required by 25% of the elderly as compared to 3.5% of the young (p=0.005). Most common comorbidities were hypertension (43.8%), diabetes (25.7%), and cardiac disease (16.2%) which were present in the 69.5% of the elderly population. The disease severity was higher in elderly owing to comorbid conditions. Similar trend was observed in the current study.

Kunno J et al.^15^ compared data of Covid19 patients in the first three waves in Thailand. During first wave, total confirmed cases were 2455 with mortality 60 (2.44%), followed by 23312 confirmed cases with 34 (0.001%) deaths during second wave and 134025 confirmed cases with 937 (0.007%) deaths during the third wave. Their number of cases increased in the successive waves, results similar to those observed in current study. However, increased mortality was observed in current study as compared to the study by Kunno J et al.^15^

A similar study was conducted by Seong H et al.^16^ in South Korea in 2021, comparing second and third wave. During second wave, total confirmed cases were 8020 with mortality 73 (0.91%) and during the third wave, total confirmed cases were 33690 with mortality 725 (1.26%). They observed increase in the number of cases and mortality rate. Current study also observed the similar trend. The rise in the number of cases can be attributed to the increased number of primary sources of infection in each successive wave.

Ramos-Rincon JM et al.^17^ studied older population in Spain. The study included 4545 cases during the first wave, and 1408 cases in the successive waves. Patients admitted in the successive waves were older with greater dependency and Charlson Comorbidity Index. Heart failure was the most common complication and increased in following waves as compared to the first wave. The case fatality rate (CFR) was significantly higher in the first wave (44.1% vs. 33.3%; p < 0.001). After adjustments to the model, the probability of death was 33% lower in successive waves. Their results contradicted with those of current study in terms of increase in the number of older cases and mortality rate in the successive waves of Covid19 pandemic.

Palmieri L et al. 18 studied Italian population during the two phases of the first wave of Covid19 pandemic. First phase was from March to May 2020 and the second phase was June to August 2020. They observed 34191 deaths in first phase and 1404 deaths in second phase. PCR positive rate was 10.3% vs. 14.5% during first and second phase, respectively. The third wave stood out with a significantly higher mortality rate compared to the other waves. Several factors may have contributed to this disparity. The overwhelming surge in cases during this period could have stretched healthcare resources and led to challenges in providing adequate care for critically ill patients 19. Furthermore, the emergence of new viral variants during the third wave may have played a role in influencing disease severity and outcomes.^20^

Freeman A et al.^21^ compared first three waves of Covid19 pandemic. Some of the parameters studied by them coincided with those of the current study. Both studied observed increased number of cases in successive waves. Serum ferritin levels were highest during the first wave as compared to the following waves. Use of Remdesivir increased in the successive waves, in both the studies. Freeman A et al.^21^ observed increase in ICU admissions and mechanical ventilator support and decrease in mortality rate in the successive waves, however, current study observed contradictory results i.e., decrease in ICU admissions and mechanical ventilator support but increase in mortality rate in the successive waves. Minchella PA et al.^22^ observed that the higher mortality was associated with the older age, as witnessed in current study.

### Limitations

This study was conducted in single hospital setting situated in a lesser developed region of the country. There is need to conduct more studies with data collection from various centers and including various clinical and biochemical parameters apart from just the number of cases and mortality rate due to Covid19.

## CONCLUSION

The findings of this study in our local context of COVID-19 pneumonia patients across pandemic waves, reveal dynamic trends in patient diseases pattern and outcomes. The initial waves exhibited higher ICU admissions and mortality rates, suggesting a need for improved early response and resource allocation. Unabated flexibility for adaptation of newer or various healthcare strategies and techniques is vital for enhancing patient care during an ever-changing pandemic landscape.

### Author`s Contributions

**MA & MHSG:** Conception and writing of manuscript, designing of methodology and analysis, data interpretation.

**AR & ZSS:** Designing of data collection tools and acquisition of data, computations and analysis, manuscript drafting.

**SH, MA & ZSS:** Conception, manuscript editing and revision for accuracy

**MA:** Responsible for the accuracy of the study.
